# Development of a training paradigm for voluntary control of the peri-auricular muscles: a feasibility study

**DOI:** 10.1186/s12984-019-0540-x

**Published:** 2019-06-14

**Authors:** Siwaphorn Chanthaphun, Sandy L. Heck, Carolee J. Winstein, Lucinda Baker

**Affiliations:** 1 Department of Biokinesiology and Physical Therapy, Health Sciences Campus, Herman Ostrow School of Dentistry, University of Southern California, Los Angeles, California 90089 USA; 2 Reach Bionics Inc., Los Angeles, CA 90048 USA; 3 Department of Biokinesiology and Physical Therapy, Health Science Campus, Herman Ostrow School of Dentistry, Department of Neurology, Keck School of Medicine, University of Southern California, Los Angeles, California 90089 USA

**Keywords:** Motor learning, Peri-auricular, Neuromuscular electrical stimulation, Biofeedback

## Abstract

**Background:**

Spinal cord injury (SCI) can lead to severe and permanent functional deficits. In humans, peri-auricular muscles (PAMs) do not serve any physiological function, though their innervation is preserved in even high level SCI. Auricular control systems provide a good example of leveraging contemporary technologies (e.g., sEMG controlled computer games) to enable those with disabilities. Our primary objective is to develop and test the effectiveness of an auricular muscle training protocol to facilitate isolated and coordinated, bilateral voluntary control that could be used in individuals without volitional control of the vestigial PAMs.

**Methods:**

Seventeen non-disabled persons were screened; 13 were eligible and 10 completed the entire protocol. The facilitation phase, included one session of sub-motor threshold, sensory electrical stimulation followed by neuromuscular electrical stimulation paired with ear movement feedback for up to 8 additional sessions. Participants progressed to the skill acquisition phase where they dawned an auricular control device that used sEMG signals to control movements of a cursor through three levels of computer games, each requiring increasingly more complex PAM coordination.

**Results:**

The 10 who completed the protocol, finished the facilitation phase in 3 to 9 sessions and achieved some level of voluntary auricle movement that ranged between 1 and 5 mm. Qualitative analysis of longitudinal post-session auricular movement, revealed two subgroups of learners. Six successfully completed all 3 games—the “Learners”. Two were partially successful in game completion and two were unable to complete a single game--“Poor/Non-Learners”. Quantitative analysis revealed a significant group difference in auricular amplitude for both facilitation and skill phases (*p* < .05), and a significant relationship between performance in the two phases (R^2^ = 0.84, *p* = 0.004).

**Conclusion:**

Sixty percent of those who completed the facilitation phase were able to learn and demonstrate functional voluntary control of the vestigial PAMs. Those who progressed the fastest through facilitation were also those who were most proficient in skill acquisition with the device. There was considerable variability in progression through the two-phase protocol, with 20% deemed Poor/Non-Learners and unable to complete even the most basic game following training. There were no serious adverse events.

**Trial registration:**

ClinicalTrials.gov Identifier: NCT02358915, first posted February 9, 2015.

## Background

Auricular control systems provide a good example of leveraging contemporary technologies (e.g., EMG signal extraction, bio-signal controlled computer games) to enable those with disabilities. Control methods such as chin drive or pneumatic systems (“sip-and-puff”) [1], tongue [2], head [3] and voice control drive systems [4] already exist. For those with a high level spinal cord injury, these various systems draw upon an already compromised and severely limited remaining motor and respiratory capacity for tasks of daily life. An alternative and perhaps more viable method of controlling both mobility and environmental systems may be the vestigial peri-auricular muscle complex which could be accessed through surface ElectroMyoGraphy (sEMG).

The peri-auricular muscles (PAMs) can be found in 95% of the population but do not currently serve any physiologic function [5–8]. Typically, three extrinsic auricular muscles comprise the peri-auricular muscle complex for each ear and are innervated by branches of the cranial nerves [6, 9, 10]. Cranial nerves do not enter or exit the spinal column, thus are unlikely to be affected in cases of SCI. If peri-auricular sEMG could be transformed into meaningful control signals, they could be used to operate or direct any device including a wheelchair or computer system.

A patented head-mount device was designed to record sEMG selectively from peri-auricular muscles. The challenge was to develop a training program to enable selective activation of PAMs. Ultimately, if successful, the PAM sEMG signal could be used to operate or direct a powered wheelchair in the way that a joystick would be used by the hand or the head. Successful application of this technology is dependent on the capacity to acquire and produce independent but coordinated and reproducible PAM contractions of each auricle. Electrical stimulation has been incorporated into muscle facilitation protocols for decades. There is considerable evidence that it provides a short-term adjunct to enhance voluntary control of specific muscles [11–13]. Electrical stimulation applied to the motor point of a muscle enhances the excitability of the motor neuron pool by partially depolarizing the motorneurons [11].

Our primary objective was to develop and test the effectiveness of an auricular muscle training protocol to facilitate isolated and coordinated, bilateral voluntary control that could be used in individuals without initial volitional control of the peri-auricular muscles. To align with the patented head-mount device, this study specifically targeted two of the extrinsic auricular muscles: the superior auricular and posterior auricular muscles bilaterally. We refer to these muscles collectively as the peri-auricular muscles (PAMs). Our long-term hypothesis is that once an individual acquires the ability to voluntarily move both of his or her ear auricles in a coordinated manner, this novel skill can be integrated through technology for reliable control of powered mobility and environmental devices.

## Methods

### Participants

This study was registered in Clinical Trials.gov (NCT02358915) and conducted at the BICE research center in the Division of Biokinesiology and Physical Therapy of the University of Southern California. Participants were recruited through campus flyers and word of mouth.

All participants were provided an institutionally approved consent form prior to initiation of the study. Inclusion criteria were: non-disabled, and without visible auricle movement on command. Exclusion criteria included: a self-reported history of traumatic brain injury, cognitive impairment, neuromuscular disease, migraine headaches, temporomandibular joint dysfunction, or seizures. Additionally, participants were excluded if they reported having an implanted pacemaker or defibrillator device or were pregnant.

### Two-phase training protocol

**Fig. 1:**
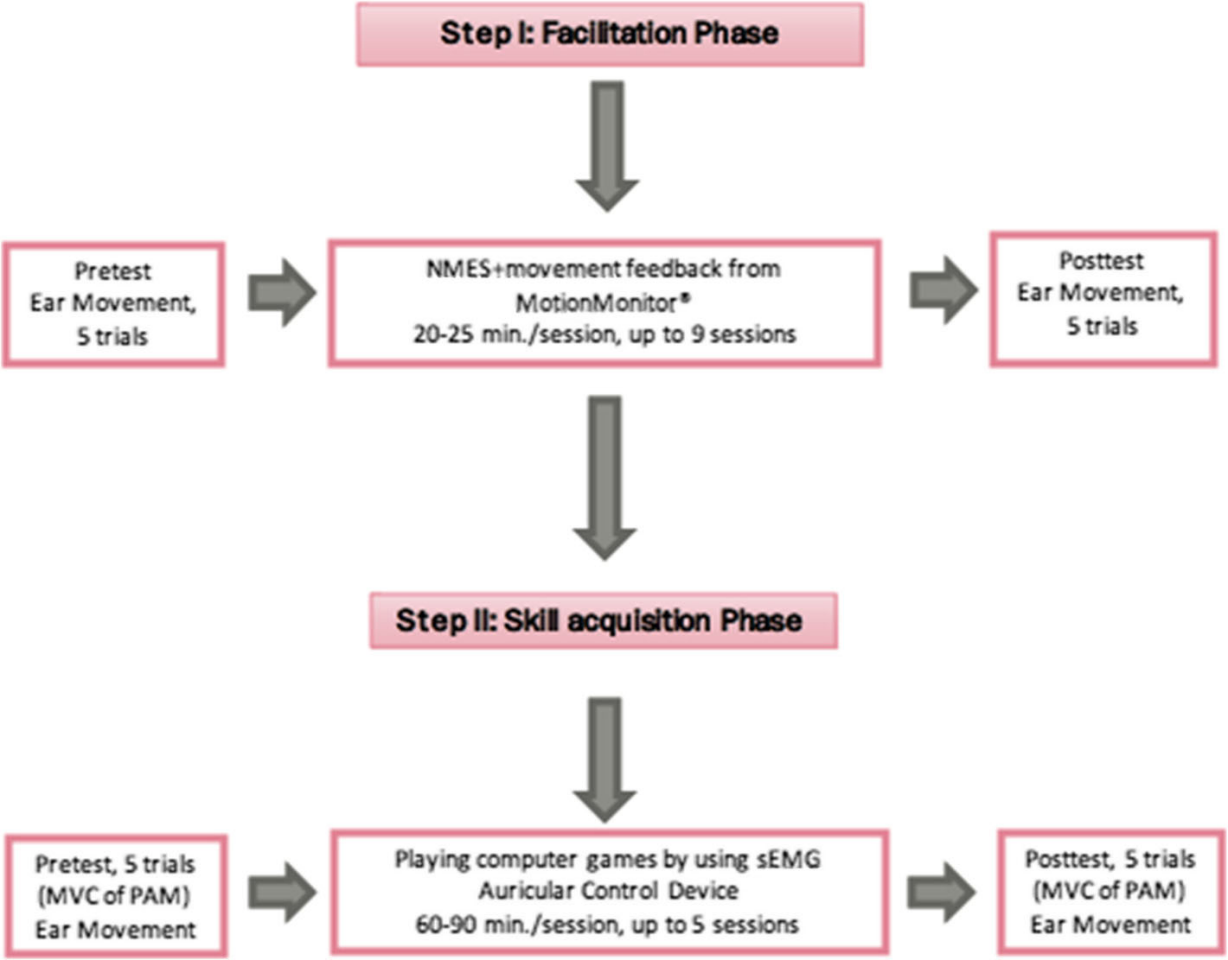
Flow Diagram Illustrating the two-phase training protocol with 5 trial pre- and post-test auricular movement performed without feedback, before and after each training phase. Phase I used electrical stimulation and ear motion biofeedback. Phase II used a four channel sEMG measurement system and software developed by ReachBionics for skill based computer game play. MVC = maximum voluntary contraction

The two-phase training protocol (Fig. 1) consisted of: Phase 1--the facilitation phase--that was designed to provide experience of PAMs location and movement through electrical stimulation and movement feedback and: Phase 2--the skill acquisition phase—that allowed participants to practice coordinated and skilled PAMs activation through three progressively more challenging goal oriented computer games while wearing the patented head-mount device. Although not required, outside of the formal training time, practice of “ear wiggling” was encouraged.

Before each practice session a 5-item questionnaire was administered to document any side effects (e.g., muscle soreness, headache) experienced after the previous session.

Our primary dependent measure is auricular movement. The maximum auricular movement without feedback was recorded before and after each training session during both phases of training (i.e., facilitation and skill acquisition) to capture learning. Specific instructions for these test sessions are to “move your ear upward and backward as far as you can and hold it for 10 seconds”.

### Technology and equipment

#### Phase 1: facilitation

The electrical stimulator was produced by FastStart VQOrtho (Irvine, CA) and is an FDA approved neuromuscular electrical stimulator. The stimulator has two independent channels for which independent stimulation levels can be set for each channel, leading to sensory level stimulation or neuromuscular level stimulation (i.e., motor level). Standard, commercially available small self-sticking electrodes (1 cm diameter, self-adhering stimulation electrodes) were placed directly on the skin over the superior and posterior auricular muscles of each ear before each training session.

**Fig. 2:**
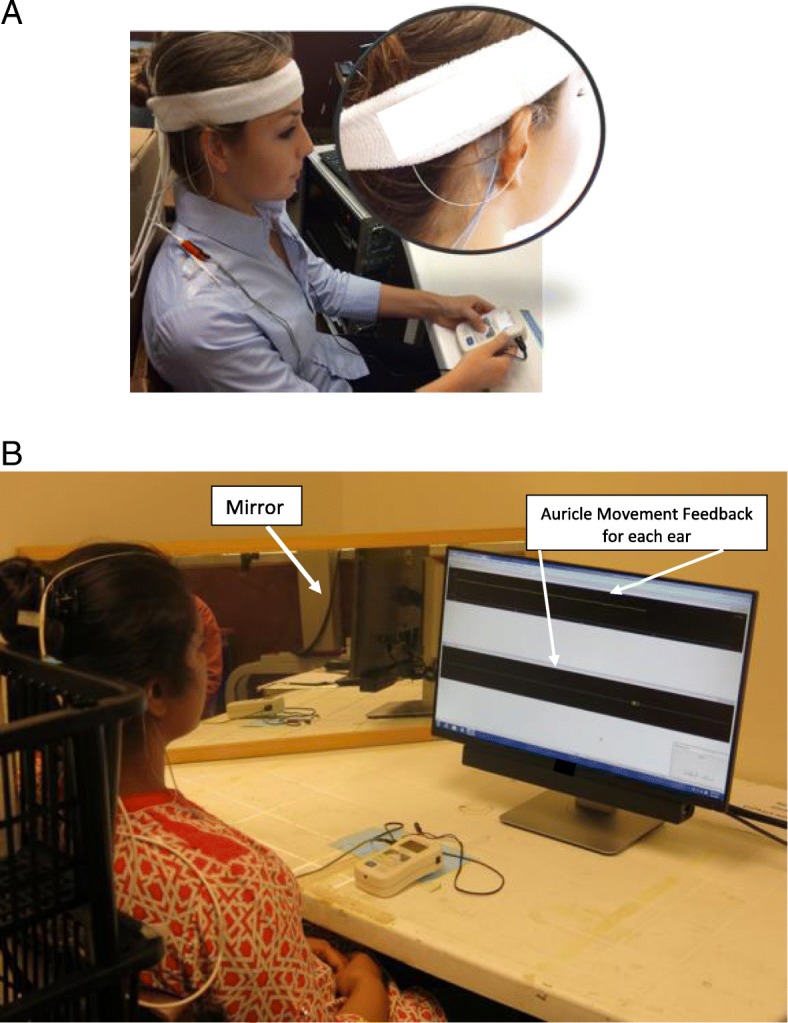
**a** The FastStart electrical stimulator held by the participant and the surface electrodes used for the facilitation phase. Electrodes were positioned on the muscle belly of the superior (not seen) and posterior peri-auricular muscles (see Insert). The insert view also shows the ear movement electrode positioned on the auricle helix that was used for motion feedback. **b** A participant attempting to activate PAMs during NMES. The computer screen with motion feedback is in front and the mirror is positioned to the left

The movement feedback was provided through the MotionMonitor® by Innovative Sports Training, Inc. version 9.22 (2015). Three sensors were placed at three locations: one on the right ear helix, one on the left ear helix, and one between the eyebrows (Fig. 2, insert). The position of the ear helix was collected through an electromagnetic marker at a sampling rate of 120 Hz throughout each testing session and provided as visual feedback on the computer screen (Fig. 2b).

#### Phase 2: skill acquisition

**Fig. 3:**
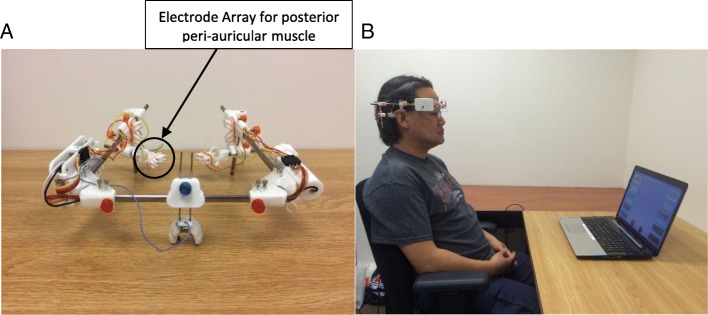
**a** the headset system contains the multi-channel non-invasive sEMG measurement system. sEMG electrodes mounted on the headset were placed on the skin around the ears over the superior and posterior peri-auricular muscles and recorded the signal using application-specific software. **b** Shows the participant with headset seated in front of the computer playing a game during a Phase II training session

Surface electromyography (sEMG) was recorded through the Biosemi Active two EMG measurement system (BATS) (BioSemi B.V., 1054SC Amsterdam, Netherlands). The head-mount device provided an array of 4 electrode contacts within each identified muscle group (superior and posterior auricular muscle, bilaterally) (Fig. 3a). Previous device testing confirmed minimal cross talk between the four channels. The electrodes worked through gold plated pins used as dry contacts on the skin surface. Figure 3a shows the head-mount device integrated onto a pair of eyeglass frames for stabilization. Figure 3b shows a participant using the head-mount sEMG acquisition system for game play.

Software was used to transform the complex coordination patterns from the four targeted peri-auricular muscles to enable control during the three computer games. The algorithm that translated sEMG to cursor control identified an “activation” in a muscle when the Root-mean-square (RMS) EMG voltage exceeded a threshold. The EMG signals were sampled at 540hz and High-pass filtered above 10hz. Recordings were taken from 8 unipolar EMG channels, paired at each of the four muscles and differentially calculated in software, ultimately providing 4 channels of output. The RMS was measured in each channel continuously over the previous 0.2 s. Activations were calculated according to ‘off’ or ‘on’ state. While a simple threshold value to determine ‘on’ state is attractive, variability in baseline noise strength and magnitude of activation strength preclude this possibility, and the threshold needed to be calculated dynamically. Thus, every 15 s, a Gaussian mixture distribution was calculated for up to 120 s of the preceding data with an assumption of two clusters (‘off’ and ‘on’ states). The current activation is then measured according to the prior probability of its corresponding to the active cluster, with a threshold of 0.9 yielding an ‘on’ state.

The system continuously performed these calculations for the four EMG channels corresponding to the four peri-auricular muscles under observation. For each of the games, a set of rules established how the game was controlled by the EMG “activations”.

In the first game (basic skill), there were 50 bricks (arrayed in a 10 × 5 grid) on the top of the screen. The participant needed to “break” them all by repeatedly bouncing the ball towards the bricks with the paddle, which they move left to right to be positioned under the ball as it bounces back. Therefore, participants must move the paddle right or left to stop a ball that is travelling at varying speeds and angles. In this game, the paddle moves if one or both of the auricular muscles on a given side are activated in the absence of an activation on the opposing side. This game required the ability to switch control from side to side.

In the second game (intermediate skill), the subjects are asked to move a cursor around a 2-D screen aiming at different targets arrayed around a circle. In this game, the cursor moves forward if there is bilateral PAM activation. The cursor turns its orientation to the right or left if one or both of the muscles on a given side are activated in the absence of an activation on the opposing side. Performance was scored using a Fitt’s law score, which is a standard metric for assessing the accuracy of a pointing device. This game required isolated and coordinated activation of all four PAMs.

In the third game (high skill), the learner is asked to fill one of four squares, each associated with a different peri-auricular muscle. In this game, a given square will only fill up if the associated muscle is activated in the absence of any other muscle. This game required selective activation of each of the four muscles without the other three. It was acceptable for the other squares to be partially filled, as long as the target square was completely filled before any other square was completely filled. The desired outcome is reflected by the learned capability for isolated muscle activation and coordination that is required for success on game 3.

### Procedure

#### Phase 1: facilitation

Phase 1 provided participants with input and experience to obtain voluntary control of PAMs for ear movement. Electrical stimulation was applied to facilitate muscular contraction. Participants were positioned in front of a mirror and the Motion Monitor© movement feedback screen which graphically provided information on the amplitude of ear movement in upward and backward directions (Fig. 2b).

For the first training session, the stimulator was set to sensory level stimulation without muscle contraction to enable a feeling of ear muscle activation. In contrast to the remaining sessions, for session 1, there was no explicit instruction to move the ears. Instead, the participant was asked to pay attention to the location of the sensory stimulation. Sessions 2 through 9 were to enhance voluntary movement of the PAMs through application of motor level neuromuscular electrical stimulation (NMES) with feedback provided about the magnitude of ear movement. Participants were asked to maximally contract in synchrony with the NMES stimulation cycle. Participants practiced for 1 min trials, up to 25 trials per session per ear, with approximately 10 s of rest between trials. Electrical stimulation parameters were set to optimize neuromuscular activation and are detailed in Table 1 [10].

**Table 1:** Electrical stimulation parameters for the facilitation phase

	Sensory Electrical Stimulation	Neuromuscular Electrical Stimulation
Frequency	80 pps	50 pps
Pulse Duration	100 μ	200 μ
Waveform	Asymmetric Biphasic	Asymmetric Biphasic
Ramp up/down (seconds)	2/2	1/0
“On”/“off” phase (seconds)	8/8	7/5

Consistent with the development of a working training protocol, we established a few guidelines for progression from facilitation to skill acquisition. Our initial assumption was that a given magnitude of ear movement would be associated with sEMG recruitment and coordination. In hindsight, this was overly simplistic. Through our analysis, we realized that ear movement was not an adequately sensitive measure of sEMG recruitment or coordination. The initial protocol was written such that we expected participants would progress to skill acquisition if they could produce 5 mm of measurable auricular movement. During protocol development, some participants demonstrated visible and consistent sEMG signals in subsequent sessions prior to or ever producing 5 mm of measurable movement. However, the only objective measure of auricular control was movement amplitude. Thus, the protocol was loosened considerably to allow for progression if: 1) visible inspection of pre-test sEMG of each new training session approximated the post-test of the previous training session; 2) there was 5 mm of measurable auricular movement, or 3) a compelling argument could be made for progression (e.g., game scenario would be more motivating and engaging for learning). This allowed us to move participants at their own pace, much like the participant-customized progression used when attempting to learn a novel skill. In addition, it acknowledged that amplitude of auricular movement might not be the best indicator of readiness to begin the skill phase of practice. See ID 7’s data for an example.

#### Phase 2: skill acquisition

The skill acquisition phase allowed participants to develop their new found volitional control by motivating practice through three progressively more challenging computer games. The sEMG signal was used to play goal-oriented computer games. Generally, practice with the games transpired over 5 sessions, each up to 1.5 h per session and for 2–3 times per week. As in the facilitation phase, participants were encouraged to practice ear movements outside of training sessions. The computer games provide a goal-oriented task for practice of bilateral PAM control, with each game increasing in difficulty.

While playing these computer games in sequence, participants wore the head-mount sEMG system shown in Fig. 3b. Graphical displays and auditory tones allowed the participant to appreciate variations in their voluntary control. This feedback was thought to enhance skill acquisition for aspects of voluntary coordination of the four peri-auricular muscles.

### Statistical analysis

Descriptive statistics were derived from the post-test auricular movement data collected after each session of the facilitation and skill phase. Means and SDs were computed from the 5 post-test trials obtained after each session. Group comparisons were performed using the non-parametric Welch’s t-test between Learners and Poor/Non-learners. All statistical tests were performed using R version 3.4.

## Results

Participants were non-disabled individuals who initially, were not able to produce visible voluntary movement of either auricle on request. A total of 17 participants were screened by phone. Thirteen were eligible and 10 completed the entire protocol. The 13 enrolled participants ranged in age from 24 to 42 years. Participant characteristics are detailed in Table 2. Participant 1’s data were unusable because of equipment malfunction. Participant 3 trained in only 1 session (sensory level stimulation) and never returned for the remainder of the study. Participant 9 experienced scheduling conflicts over the first four training sessions and withdrew from training.

**Table 2:** Participant Characteristics (*n* = 13)

Characteristics	N (13)
Age (years) Mean ± SD	29.00 ± 5.34
Age Range (years)	24–42
Gender	
Female	5
Male	8
Hand Dominance	
Right	13
Occupation	
Student	11
Other	2

**Fig. 4:**
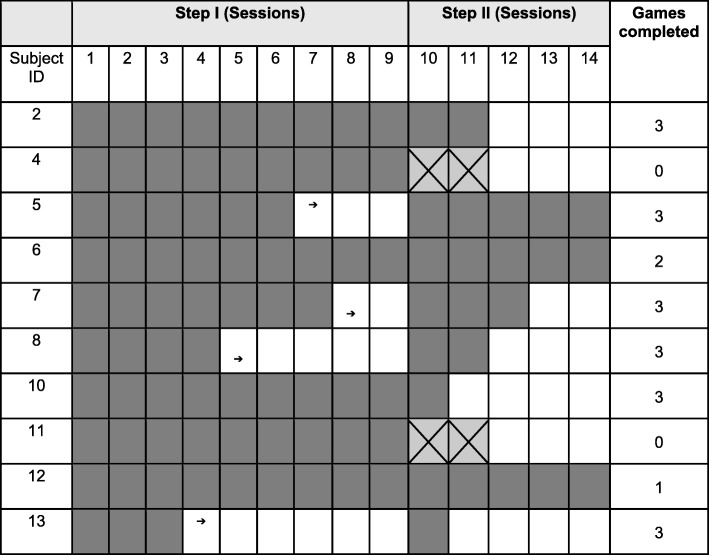
Progression trajectory for each of the 10 participants in the facilitation and skill acquisition phase. Each row is for a different participant labelled with the subject ID. Dark shading indicates successful completion of training session. Arrows depict early progression to the skill acquisition phase. “X” indicates no progression through the computer games. The last column is a tally of the number of games completed out of 3 for each participant

The ten remaining participants completed the facilitation phase ranging from 3 to 9 sessions. Six out of ten (ID # 2,5,7,8,10,13) took between 1 and 5 sessions to successfully complete the skill acquisition phase (i.e., progressed through all 3 games). We term this group the “Learners”. Of the four remaining participants, two were partially successful in skill acquisition (ID# 6,12), that is they progressed through fewer than three, but not zero games. Two participants attempted two sessions of skill acquisition, but were unsuccessful (ID# 4, 11). We label these four participants the “Poor/Non-Learners”. Figure 4 summarizes the session progression trajectory for each of the 10 participants through the facilitation and skill acquisition phases along with the primary outcome (i.e., games completed) listed in the last column. The six Learners completed all 3 games, while the Poor/Non-Learners, completed fewer than 3 games.

**Fig. 5:**
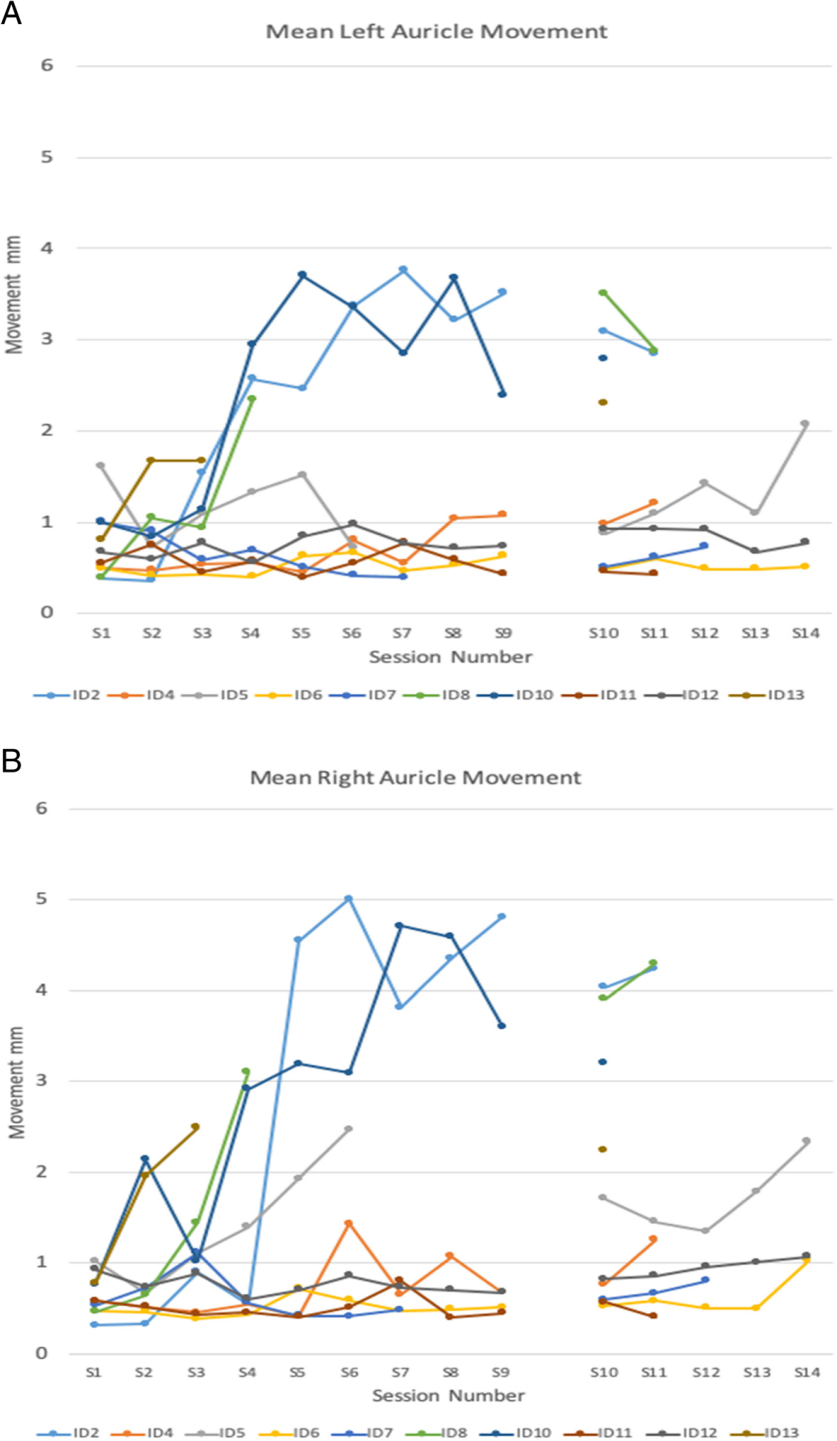
Post-test mean auricular movement (mm) by session for the left (**a**) and right (**b**) auricle for each of the 10 participants who completed the full protocol. These test measures were taken shortly after completion of each session and without feedback. Attempts at ear motion are shown following each facilitation phase session on the left (sessions #1–9), and ear motion after each skill acquisition session are shown on the right side of each plot (session 10–14).The number of sessions varied between participants as reflected by the individual line plots. For example, participant #13 (Learner) experienced 3 facilitation sessions and was successful in all three games during the first skill acquisition session (seen as a singular symbol for session 10). This is in contrast to participant # 6 (Poor/Non-Learner), who had a full complement of 14 training sessions (9 facilitation and 5 skill acquisition) but succeeded in only 2 out of 3 games

Figure 5 shows individual participant post-test mean auricular movement from each ear for the facilitation phase (session 1–9) and the skill acquisition phase (session 10–14). We used this auricular movement as a proxy for learning. Learning was defined as the capability to volitionally activate PAMs to move the auricle. The post-test was a good proxy for learning because it was acquired systematically after each training session without feedback and it resembled a standard retention test (see Fig. 1).

There is considerable variability across participants in mean movement amplitudes within auricle but not much difference within participant between auricles. We therefore averaged the mean (SD) across auricles for the data in Table 3 for the 9 facilitation sessions. For some of the participants, there is a clear increase in auricular movement amplitude as they progress through the facilitation phase. But for others, there is very little change with amplitude hovering below 1 mm. This same pattern can be seen in the data plotted in Fig. 5. Five of the six Learners demonstrate greater than 2 mm of movement during the skill phase in both auricles. The exception to this pattern is ID# 7, who was categorized as a Learner, but never achieved more than 1 mm movement in either auricle.

**Table 3:** Mean (SD) ear movement for the 9 facilitation sessions by participant

ID	1	SD	2	SD	3	SD	4	SD	5	SD	6	SD	7	SD	8	SD	9	SD
S2	0.35	(0.05)	0.34	(0.04)	1.22	(0.63)	1.56	(0.23)	3.50	(0.40)	4.19	(0.29)	3.79	(0.18)	3.78	(0.28)	4.16	(0.15)
S4	0.54	(0.09)	0.49	(0.07)	0.49	(0.04)	0.55	(0.09)	0.43	(0.05)	1.12	(0.18)	0.60	(0.06)	1.05	(0.25)	0.87	(0.10)
S5	1.32	(0.22)	0.70	(0.22)	1.10	(0.24)	1.36	(0.39)	1.72	(0.41)	1.59	(0.23)	NA	NA	NA	NA	NA	NA
S6	0.48	(0.07)	0.44	(0.04)	0.41	(0.03)	0.41	(0.04)	0.67	(0.07)	0.63	(0.07)	0.47	(0.07)	0.51	(0.08)	0.57	(0.04)
S7	0.77	(0.07)	0.81	(0.16)	0.85	(0.14)	0.62	(0.05)	0.46	(0.08)	0.41	(0.04)	0.44	(0.07)	NA	NA	NA	NA
S8	0.43	(0.04)	0.84	(0.10)	1.19	(0.62)	2.72	(0.75)	NA	NA	NA	NA	NA	NA	NA	NA	NA	NA
S10	0.88	(0.21)	1.49	(0.44)	1.08	(0.44)	2.93	(0.38)	3.45	(0.27)	3.23	(0.88)	3.78	(0.27)	4.13	(0.22)	3.00	(0.43)
S11	0.57	(0.17)	0.63	(0.13)	0.44	(0.03)	0.51	(0.13)	0.40	(0.04)	0.53	(0.10)	0.79	(0.11)	0.49	(0.11)	0.44	(0.05)
S12	0.80	(0.16)	0.67	(0.06)	0.83	(0.06)	0.58	(0.05)	0.77	(0.12)	0.92	(0.12)	0.75	(0.12)	0.71	(0.07)	0.71	(0.08)
S13	0.79	(0.10)	1.82	(0.48)	2.08	(0.14)	NA	NA	NA	NA	NA	NA	NA	NA	NA	NA	NA	NA

Units are mm of auricle movement recorded at the post-test without feedback for each session. These Means and SD are averaged across the two ears. NA = not available, participant completed the facilitation phase and had advanced to the skill phase. Subject ID is consistent across all Tables and Figures. Learners were ID 2, 5, 7, 8, 10, and 13. Poor/Non-learners were ID 4, 6, 11, and 12

**Fig. 6:**
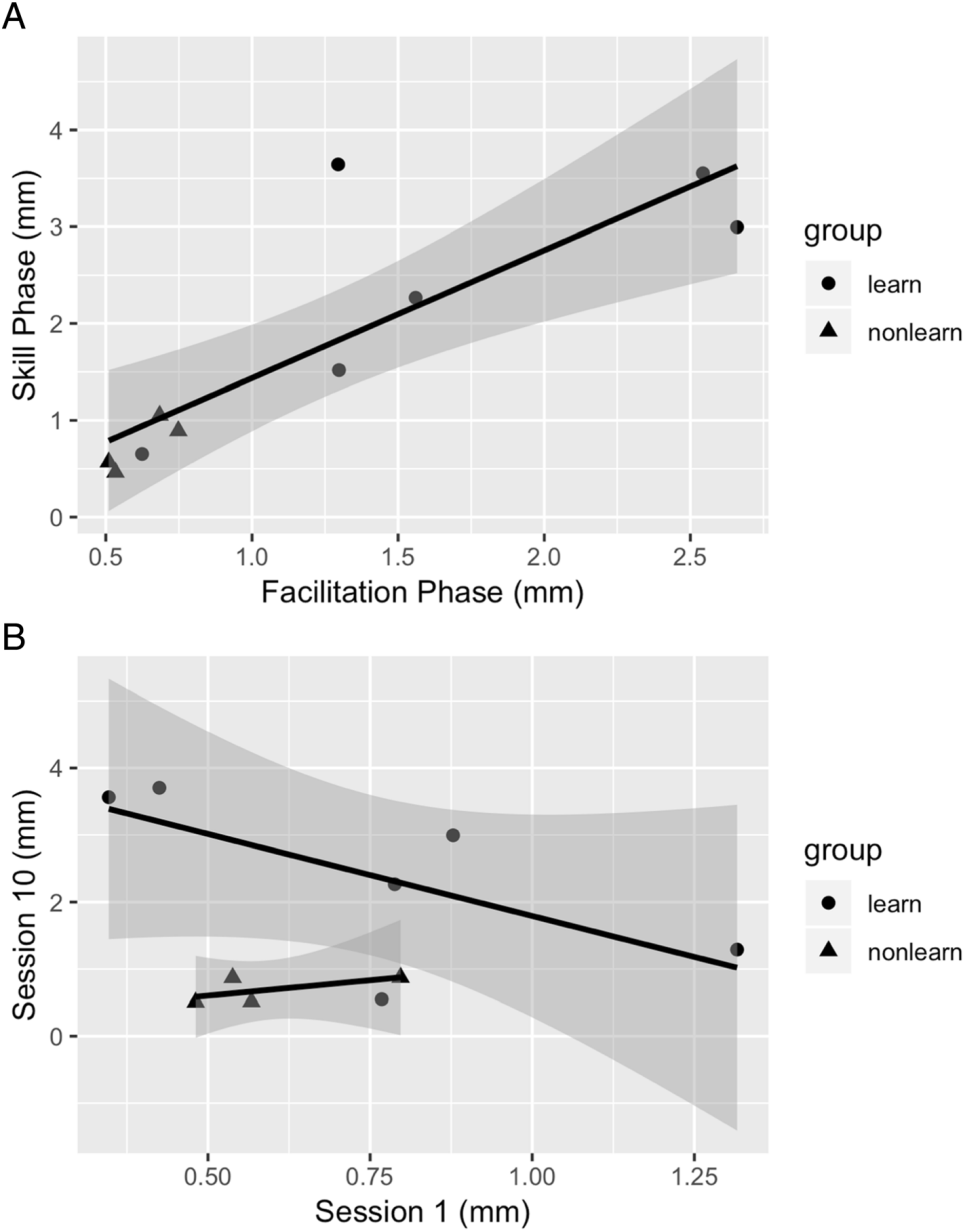
**a**. Scatterplot of the relationship between mean auricular movement for the facilitation phase and the mean auricular movement achieved on the post-test sessions for the skill acquisition phase for Learners (solid circle) and Poor/Non-learners (solid triangle). Spearman’s rank correlation coefficient, R^2^ = 0.84, *p* = 0.004. **b**. Scatterplot of the relationship between mean auricular movement on the post-test after the first facilitation session (sensory session 1) and the first skill acquisition session with the games (session 10). Notice two different linear fits—one for Learners (solid circles) and one for Poor/Non-learners (solid triangles). Note that ID# 7 (a Learner, solid circle) is clustered among the Poor/Non-learners (solid triangles)

Quantitative analyses (Welch’s t-test) between Learner and Poor/Non-Learner groups revealed significant group differences in mean auricular amplitude for both facilitation and skill phases (Table 4, *p* < .05). Figure 6a shows a scatterplot of Learners and Poor/Non-Learners revealing a significant linear fit between mean auricular amplitude for the facilitation and skill phases (R^2^ = 0.84, *p* = 0.004). Notice the lone Learner (ID#7, solid circle) clustered at the low end of the linear fit line among the Poor/Non-Learners (solid triangles) with amplitudes <1 mm. Figure 6b is a scatter plot showing the relationship between the first session of the facilitation phase (sensory session) post-test, and the first session of the skill phase post-test—first experience with game play. Notice there are two linear fit lines—one for Learners and one for Poor/Non-learners. We are cautious about reading too much into this scatterplot because of the small sample size, however, again ID#7 is clustered among the Poor/Non-learners. This observation highlights the difference between auricular movement and sEMG motor control. Two of the Poor/Non-Learners (ID# 6, 12), were given 5 sessions to demonstrate skill, but in spite of achieving a rudimentary level of control, were not able to progress to the more selective control required for game 3. The two others in the Poor/Non-Learner group (ID # 4,11) exhibited less than 2 mm of movement and inconsistent levels of auricular movement with each ear.

**Table 4:** Mean and SD ear movement (mm) from Learners and Poor/Non-learners in the facilitation and skill Phase, and the 95% CI of their mean difference. Estimates from Welch’s t-test and associated degrees of freedom (df) and *p*-values

	Learners		Poor/Non-Learners		95% CI of Mean Difference		*t*-value	Df	*p*-value
	*Mean*	*SD*	*Mean*	*SD*	*Lower*	*Upper*			
Facilitation Phase	1.66	0.79	0.62	0.12	0.22	1.87	3.18	5.32	0.022*
Skill Phase	2.44	1.19	0.74	0.27	0.45	2.94	3.36	5.77	0.016*

*statistically significant at *p* < 0.05

No serious adverse events were reported by any of the participants. Side effects of training were reported in the 5-item questionnaire as mild headaches (*N* = 4), muscle soreness (*N* = 3) and fatigue (*N* = 2). The majority of these side effects occurred during the initial training sessions. None of these reported symptoms prevented or deterred participants from continuing training and resolved within one to two sessions during the facilitation phase.

## Discussion

The primary objective of this feasibility study was to develop and test the effectiveness of an auricular muscle training protocol to facilitate isolated and coordinated, bilateral voluntary auricular motor control that could be used in individuals without initial volitional control of the peri-auricular muscles. To date, very little research exists regarding how one would train volitional activation of the PAMs. Schmalfuß and colleagues demonstrated that non-disabled participants and individuals with tetraplegia could successfully be trained to activate the posterior peri-auricular muscles for basic steering commands [13]. Our protocol was designed to address two major limitations of Schmalfuß and colleagues. First we recruited individuals without initial PAM control—those unable to wiggle their ears. Second, we trained two muscle pairs (bilateral superior and posterior peri-auricular muscles) instead of just one pair, to better approximate the complex demands required for environmental control.

Uniquely, we combined NMES, a well-known facilitation tool [14, 15] and the Auricular Control Device that used sEMG as an input to control cursor movements for progressively more complex game scenarios. We reasoned that an initial facilitation phase prior to functional training may be more likely to enhance motor learning and skill acquisition reflected by skilled motor control and coordination such as that seen for a majority of participants in this study.

Qualitative and quantitative analyses of post-test auricular movement data revealed two distinct groups. In general, the Learners (*N* = 6) demonstrated increased auricular movement performance over sessions and the ability to translate that capability into functional motor control---essential for game success. There was one exception--ID #7 did not demonstrate auricular movement greater than 1 mm, but went on to demonstrate sEMG control and coordination sufficient to succeed at the most challenging game and accomplished this level of skill quickly in only 3 game play sessions out of 5. This illustrates the difference between measurable goal-directed auricular movement (post-test performance) and implicit sEMG activation in the game context that we noted earlier. Indeed, this participant may have benefited from the well-known external focus of attention strategy highlighted through game play where the focus is directed at the movement effect (i.e. move the cursor) and not directed at the performer’s body movements (i.e. move the auricle) [16].

Common to the Poor/Non-Learner group (*N* = 4) was inconsistent auricular movement performance and limited movement amplitude demonstrated both within and between auricles across sessions. For these participants, we suspect that training of each ear, independently, did not transfer well into coordinated control of both auricles—an essential requirement for success in the most difficult game. This has important implications for the eventual training of the vestigial peri-auricular muscle complex for controlling both mobility and environmental systems with sEMG. The other two participants in the Poor/Non-Learner group demonstrated consistently poor movement ability (i.e., less than 1 mm) after nine facilitation sessions. Even after two sessions in the more implicit and engaging game scenario, the Poor/Non-Learners were unable to achieve the most basic form of coordination (game 1-switching between right and left PAMs.) For these participants, the initial facilitation phase did not seem to help them find the muscle and establish basic volitional control. We cannot rule out the possibility that after training over more sessions, these two (ID# 4, 11) would have acquired the skill.

### Limitations

Several important limitations are acknowledged. First, we did not have a control group attempt to use the Auricular control device without participation in the sensory and motor facilitation phase. As such, we cannot effectively evaluate if the facilitation phase benefited the skill learning over simple practice with the game device. Second, we used post-test auricular movement amplitude as a proxy for skilled control instead of game performance data. Third, we did not systematically track outside of lab self-practice. Such data would reflect additional practice that may have impacted the rate at which our participants acquired (or did not acquire) the skill. Forth, generalization of these findings are limited by the fact that we enrolled non-disabled participants only. However, the targeted population of individuals with high tetraplegia would likely have intact neural circuitry for PAM along with lesion-associated cortical reorganization that could serve to amplify the effectiveness of this training protocol. Future studies are needed to better model the learning of PAM control and to predict learners and non-learners using a combination of sEMG, auricular movement control measures and other factors such as outside of lab self-practice.

## Conclusions

Sixty percent of those who completed the facilitation phase were able to learn and demonstrate functional voluntary control of the superior and posterior peri-auricular muscles. Those who progressed the fastest through the facilitation phase (i.e. fewer sessions) were also those who were most proficient in the skill acquisition phase. There was considerable variability in progression through the two-phase protocol, with 20% of the participants deemed Poor/Non-Learners and unable to complete even the most basic game following a full-compliment of facilitation phase training.

## Data Availability

ClinicalTrials.gov Identifier: NCT02358915. First posted February 9, 2015. The data set obtained during the study is available from the corresponding author upon reasonable request.

## Abbreviations

NMES: Neuromuscular electrical stimulation
PAMs: Periauricular muscles.
sEMG: surface electromyography
